# Targeting nutrient retrieval by *Francisella tularensis*

**DOI:** 10.3389/fcimb.2013.00064

**Published:** 2013-10-14

**Authors:** Yousef Abu Kwaik

**Affiliations:** Department of Microbiology and Immunology, College of Medicine, University of LouisvilleLouisville, KY, USA

**Keywords:** tularemia, legionella, autophagy, proteasome, legionnaires disease

Our continued progress in understanding microbial pathogenesis has been fueled, to a large extent, by the discovery of various translocation systems that inject a large cadre of eukaryotic-like and novel microbial effectors into the host cell. These effectors manipulate a myriad of host cell processes and subvert innate and adaptive immunity through novel and exciting mechanisms. The injected bacterial effectors and their host cell targets are potential candidates for anti-microbial therapy.

Nutrition and proliferation is fundamental to life, and all organisms have evolved to maximize their harvest of energy and biomass building blocks from available nutrients. This applies also to pathogens that colonize host tissues and cause disease. It is a simple logic that without proper nutritional resources for survival/proliferation in the host, bacterial pathogens do not cause disease. It can be challenging for microbial pathogens to obtain nutrients during infection, since part of the host innate defense is to restrict pathogen access to various essential nutrients (Abu Kwaik and Bumann, 2013). Successful pathogens have therefore evolved highly efficient nutrient retrieval strategies to counteract host nutritional limitation or deprivation of major sources of carbon and energy (Abu Kwaik and Bumann, 2013). Microbial acquisition of nutrients and metabolism *in vivo* is a major fundamental aspect of infectious diseases that impacts virulence, pathology, and efficacy of antibiotic treatment. Despite these simple facts, our knowledge of microbial nutrition *in vivo* still remains very limited, which is likely due to the experimental challenges in deciphering microbial nutrition and metabolism within complex microenvironments in the host. Numerous global genome-wide transcriptome and mutant screens have identified a large cadre of metabolic genes and pathways that are expressed *in vivo*, but their functional relevance remain largely unclear.

It has been widely presumed that the host cell cytosol is a rich haven of nutrients for microorganisms to proliferate in Abu Kwaik and Bumann (2013). However, recent evidence from studies on *Francisella tularensis* indicates that this presumption is short sighted (Alkhuder et al., 2009; Steele et al., 2013). After entry into mammalian and arthropod-derived cells, *F. tularensis* rapidly escapes from the acidified late endosome-like phagosome into the cytosol (Akimana and Kwaik, 2011; Asare and Abu Kwaik, 2011). While *F. tularensis* is auxotrophic for Cysteine, it is the least abundant and most limiting cellular amino acid in mammals (Price et al., in press). To counteract host limitation of Cys, *F. tularensis* exploits host glutathione (GSH) (Alkhuder et al., 2009; Meibom and Charbit, 2010), which is the most abundant source of Cys in the host cytosol (Franco et al., 2007). Glutathione is a non-ribosomal tri-peptide (L-γ-L-glutamyl-L-Cysteinyl-glycine) present in almost all eukaryotic cells and some prokaryotes, and its synthesis in eukaryotes is limited by the relatively low levels of cellular Cys (Franco et al., 2007). *F. tularensis* utilizes the enzyme γ-glutamyl transpeptidase (Ggt), which cleaves GSH to liberate Cys, raising the cellular levels of Cys needed to power intracellular proliferation in the host cell cytosol (Alkhuder et al., 2009; Meibom and Charbit, 2010). The severe intracellular growth defect of the *ggt* mutant of *F. tularensis* is rescued by supplementation of Cys (Alkhuder et al., 2009). In addition, requirement for high levels of Cys for *in vitro* growth of *F. tularensis* is bypassed by supplementation of GSH (Alkhuder et al., 2009). Therefore, Ggt should constitute a promising target for anti-microbial therapy to block access of *F. tularensis* to a rich source of an essential amino acid for intracellular proliferation.

Recent studies on the two intra-vacuolar pathogens, *Legionella pneumophila* (Price et al., 2011) and *Anaplasma phagocytophilum* (Niu et al., 2012), have indicated that the host cell cytosol does not have sufficient levels of amino acids needed to support the robust proliferation of these intra-vacuolar pathogens. To counteract this host limitation, the two pathogens target two major host degradation pathways to generate a surplus of amino acids to meet their demands for biomass buildup during their robust intracellular growth. While *L. pneumophila* promotes host proteasomal degradation (Price et al., 2011), *A. phagocytophilum* triggers the host autophagy degradation pathway (Niu et al., 2012). In either case, blocking the host degradation machinery blocks intracellular proliferation of the pathogen, and the block is relieved upon supplementation of excess amino acids (Price et al., 2011; Niu et al., 2012). In the case of *L. pneumophila*, supplementation of Cys, or Ser, pyruvate, or citrate also bypasses the requirement of host proteasomal degradation for intracellular proliferation (Price et al., 2011). This confirms that host proteasomal degradation provides carbon and energy sources to support the robust intra-vacuolar proliferation of the pathogen. It is possible that intra-vacuole pathogens may be inefficient in their retrieval mechanisms of host amino acids from the cytosol into their vacuoles. However, recent studies on *F. tularensis* supports the idea that the levels of host cell amino acids are simply not sufficient to support the high demands for carbon and energy to support the robust intracellular proliferation of bacterial pathogens that rely on amino acids as major sources of carbon and energy (Alkhuder et al., 2009; Steele et al., 2013).

To meet the demands for high levels of amino acids for its robust growth in the host cell cytosol, *F. tularensis* triggers the host macroautophagy degradation machinery, which is required for optimal intracellular bacterial growth (Steele et al., 2013). Similar to the approach used in *L. pneumophila* (Price et al., 2011), inhibition of the host degradation machinery blocks intracellular growth, but the block is relieved upon supplementation of excess amino acids or pyruvate (Steele et al., 2013). Interestingly, *F. tularensis* triggers a non-canonical ATG5-independent autophagy pathway that induces degradation of cellular proteins, which is predicted to generate a surplus of amino acids to support the robust intracellular bacterial replication (Steele et al., 2013). It would be interesting to identify the bacterial factor responsible for triggering host autophagy, as this would constitute a promising potential target for anti-microbial therapy to block intracellular proliferation of *F. tularensis* and manifestation of tularemia. It is also possible that the ATG5-independent autophagy pathway, which is triggered by *F. tularensis*, would constitute a potential target to prevent the infection and manifestation of the disease. One may also think that multi-targets of nutrient retrieval strategies that include Ggt and the bacterial autophagy inducer would constitute a strong approach for treatment of tularemia. As we continue to unravel mechanisms of nutrient retrieval by *F. tularensis* and other intracellular bacterial pathogens, we continue to expand our anti-microbial potential and promising targets to prevent tularemia and other infectious diseases for which no effective antibiotics are available any more.
